# Negative pressure wound therapy and split thickness skin graft aided in the healing of extensive perineum necrotizing fasciitis without faecal diversion: a case report

**DOI:** 10.1186/s12893-018-0411-6

**Published:** 2018-09-24

**Authors:** Yuan Tian, Ting Liu, Chuan-qi Zhao, Ze-yuan Lei, Dong-li Fan, Tong-chun Mao

**Affiliations:** Department of Plastic and Cosmetic Surgery, Xinqiao Hospital, Army Medical University, Xinqiao Road, Sha Ping Ba District, Chongqing, 400037 People’s Republic of China

**Keywords:** Perineum necrotizing fasciitis, Fournier gangrene, Negative pressure wound therapy, Skin graft, Faecal diversion

## Abstract

**Background:**

Perineum necrotizing fasciitis, also known as Fournier gangrene (FG), is a rare but highly mortal infectious necrotizing fasciitis with or without involvement of the underlying muscle. Evidence exists that negative pressure wound therapy (NPWT) combined with a split thickness skin graft (STSG) can help to heal wounds with FG. However, when the wound spreads to the anal area, it can easily be contaminated by faeces, causing a more extensive wounds; thus, faecal diversion is considered. Here, we report a case of extensive perineum necrotizing fasciitis that spread to near the anus; NPWT combined with STSGs was used to help heal the wound without faecal diversion.

**Case presentation:**

A 47-year-old male patient was admitted with extensive perineum fascia necrosis caused by *Pseudomonas aeruginosa* that rapidly spread to near the anus. After comprehensive therapy completed wound bed preparation, STSGs from the scalp were grafted to the wound, and NPWT was applied to improve STSGs survival and seal the anus without faecal diversion.

After treatment, graft take was 95%, and the exposed testicular and residual wounds were repaired with a local skin flap. At discharge, the wound had decreased to two pea-sized areas. The patient received conventional moist gauze therapy to close the residual wound at the local hospital. A follow-up by telephone 1 month later showed that both wounds had healed and that the patient was satisfied with the outcome.

**Conclusion:**

NPWT use combined with STSGs to cover the whole wound and the anus without faecal diversion is a safe and effective method to help with wound healing and avoid contamination with excrement.

## Background

FG is a rare but highly infectious necrotizing fasciitis of the perineum and genital region, with or without involvement of the underlying muscle [1].This condition represents a surgical emergency and has a high mortality rate, ranging from 0 to 67% [2]. Due to the rapid progression of the disease, an early diagnosis of FG is vital to identify the need to start appropriate treatment. The principles of management are achieving haemodynamic stability, the use of broad-spectrum antibiotics, prompt surgical debridement, and the positive prevention of complications [3, 4]. Some studies have shown that NPWT can secure STSGs and improve graft survival in the treatment of FG [5]. However, when the infection spreads to near the anus, the wound is prone to faecal contamination [1, 6]. Few reports have described the use of NPWT combined with STSGs in the anus area while also considering faecal contamination.

## Case presentation

**Fig. 1:**
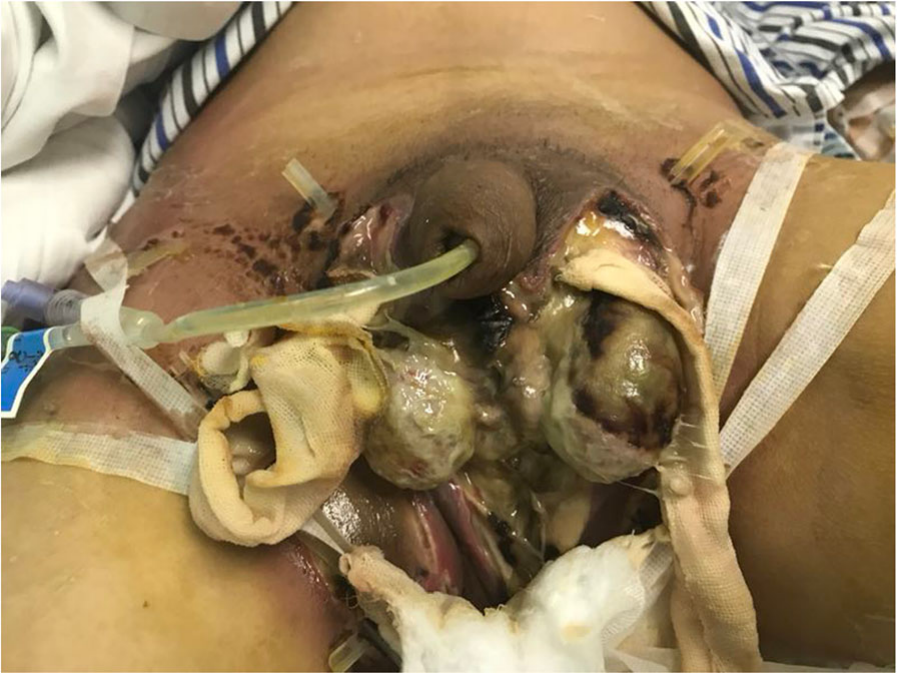
The total scrotal skin defect, extensive left and right inguinal region skin defects (4 × 10 cm and 5 × 15 cm, respectively), and purulent necrotizing tissues were covered; these were accompanied by swelling and erythema in the surrounding skin. Bilateral testes were exposed. Five drainage tubes are visible

A 47-year-old previously healthy male patient presented with a paroxysmal and progressively scrotal ache and a bilateral inguinal region with a burning sensation. He was admitted to the local hospital 4 h after onset. During admission, a suspected diagnosis of scrotitis was made, and the patient then underwent emergent and extensive surgical debridement. Past-operative histology showed chronic suppurative inflammation. However, despite the treatment, the skin of the scrotum continued to necrotize, and he was transferred to our hospital immediately. Physical examination showed a total scrotal skin defect, extensive left and right inguinal region skin defects (4 × 10 cm and 5 × 15 cm, respectively) and a purulent necrotizing tissue covering, which was accompanied by swelling and erythema of the surrounding skin. Bilateral testes were exposed. Five drainage tubes were visible(Fig. 1). The patient remained afebrile with stable vital signs. Blood chemistry showed a white blood cell count of 10.35 × 10ˆ9 /L (neutrophil percentage 86.5%), a red blood cell count of 1.18 × 10ˆ9 /L, an albumin count of 26.8 *g*/L, and an erythrocyte sedimentation rate count of 84 mm/h. An ultrasound scan showed that the necrotic scrotal wall was thickened with edema, internal echo heterogeneity, a hydrocele of the right tunica vaginalis, and normal blood supply to the testes and epididymis. Cultures of the exudates from the scrotal wound grew *Pseudomonas aeruginosa*. The admission diagnosis was perineum necrotizing fasciitis. There was no history of trauma or symptoms of dysuria or haematuria. The patient had no history of diabetes, high blood pressure, or other chronic diseases; his past surgical history was unremarkable, and he was not on any regular medications.

**Fig. 2:**
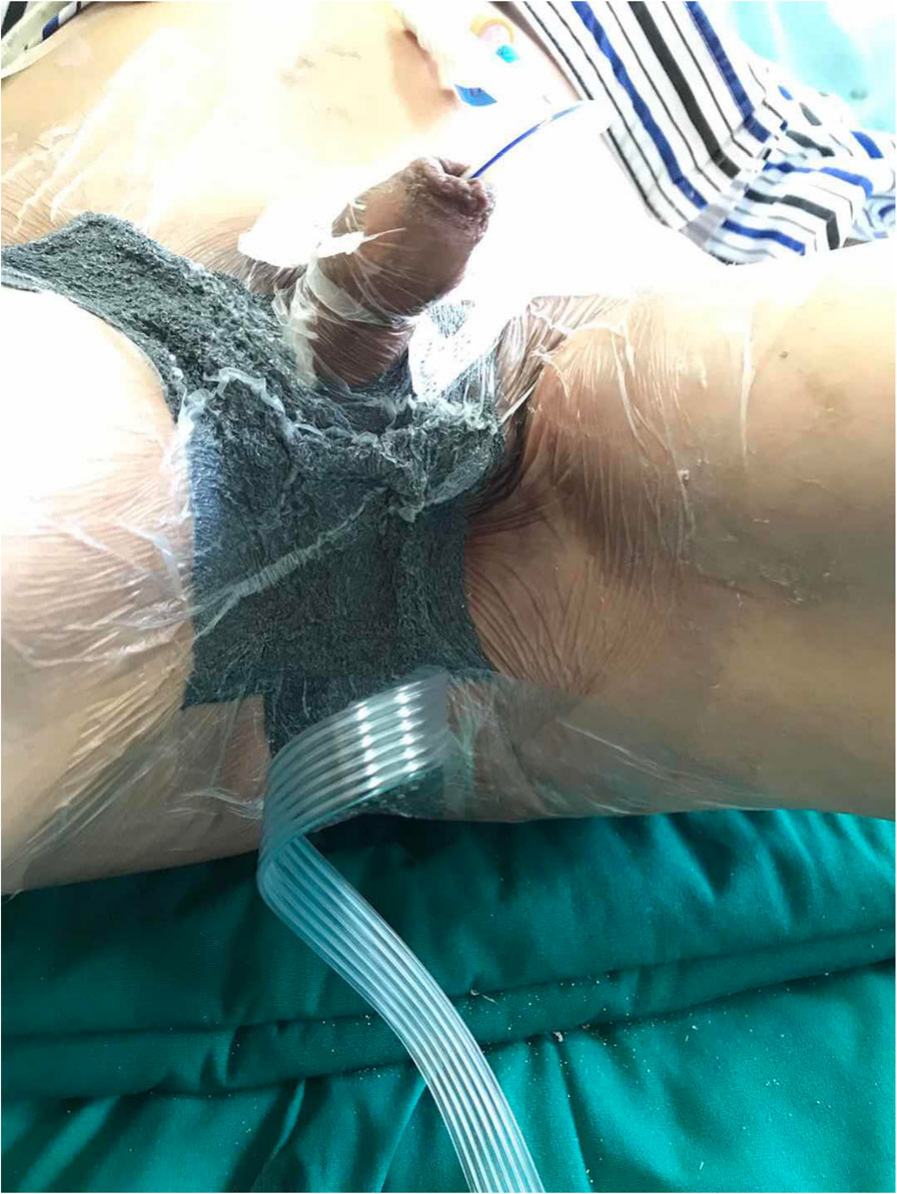
Intra-operative iodoform gauze filled the anus, a negative pressure dressing covered the wound and the anus, and a negative sucker was placed on the anus. While using the NPWT, the patient did not defecate but passed gas normally

After hospitalization, comprehensive therapy was managed by the patient. The patient then presented with fugacious pyrexia (37.8 °C), further erythema and swelling that spread to the right lower abdominal wall, which was hardened on palpation 5 days after admission. Because the patient’s condition had worsened, immediate surgical debridement was necessary. Histology confirmed the preoperative diagnosis. Cultures of exudates from the scrotal wound grew *Pseudomonas aeruginosa* and *Klebsiella pneumoniae,* and the antibiotic treatment was adjusted according to the drug sensitivity results. On the 14th day of admission, the erythema and swelling continued to extend to the right outside the groin region and 2 cm down to the perianal region. There was no other discomfort. Although the lesion might have extended, we continued to strengthen the conservative treatment rather than use surgical debridement. After extensive drug-resistance developed, the antibiotic was withdrawn and conventional moist gauze therapy was applied after a shower once or twice daily. When the wound bed had been prepared, STSGs from the patient’s scalp were grafted to the scrotum, perineum, inguinal region, and perianal region. We gave the patient intravenous Cefuroxime for 3 days after his STSG, to which he was sensitive. To secure the STSGs and improve graft survival, a vacuum sealing drainage dressing (WEGO) was placed to cover the wound and the anus, and a negative sucker was placed upon the anus for 5 days. While using the NPWT, the patient did not defecate but passed gas normally (Fig. 2). For economy, NPWT was used only after STSGs to help with wound healing. During this time, the patient did not complain of any pain or bleeding, which are common complications of NPWT. The dressing was changed every 3 days, after which the patient was able to defecate normally. After the grafted skin had survived, semi-exposure therapy was continued to facilitate wound healing. The patient had a urinary catheter placed throughout the treatment process.

**Fig. 3:**
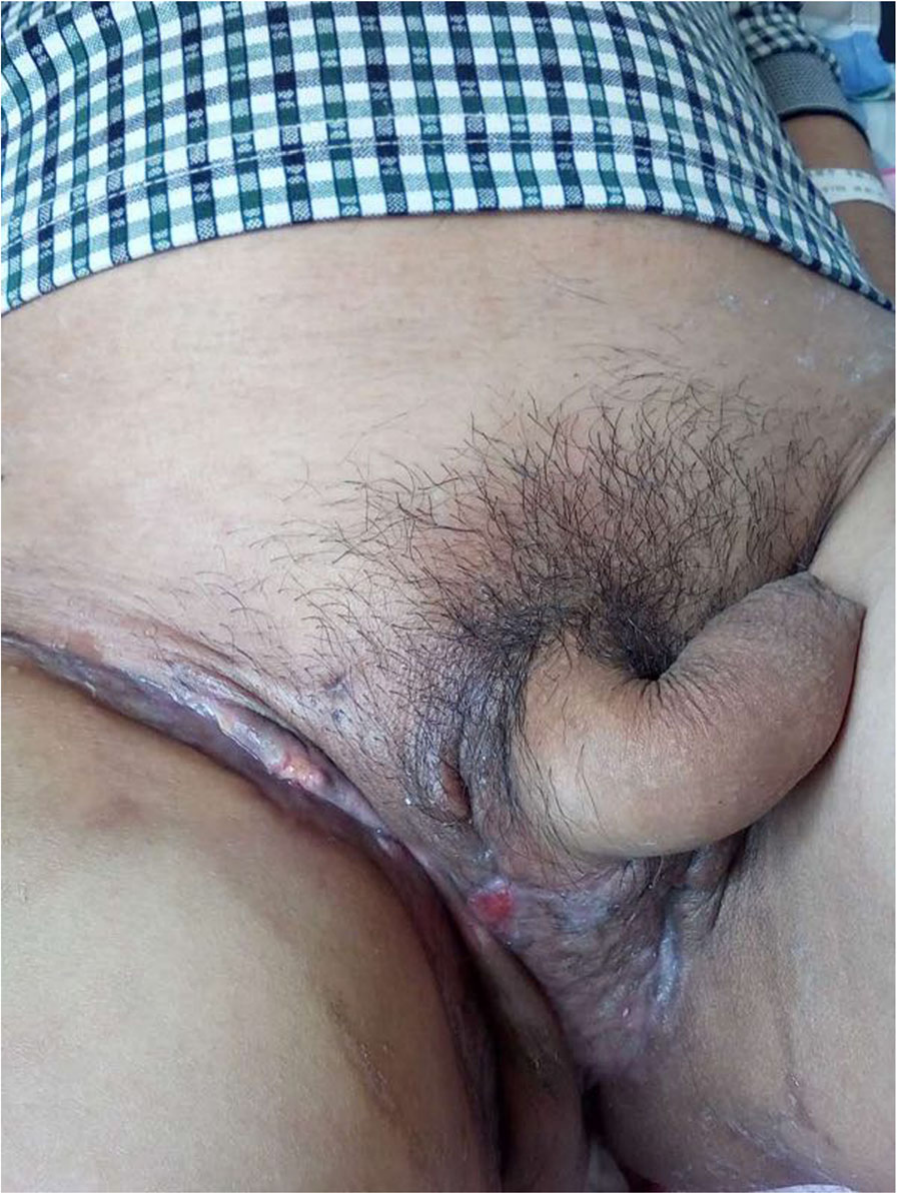
The wound had decreased in size to two pea-sized areas

After the treatment, graft take was 95%; local flaps were then grafted to cover the residual wound and the testis. The wound had decreased to two pea-sized areas (Fig. 3). The patient was discharged and received conventional moist gauze therapy to close the residual wound at his local hospital. A follow-up by telephone 1 month later showed that both wounds had healed and that the patient was satisfied with the outcome.

## Discussion

FG is an infectious necrotizing fasciitis of the perineum and genital regions that is caused by a mixture of aerobic and anaerobic organisms [1, 7]. When the skin barrier is breached, the organisms appear to spread into the subcutaneous tissue and produce fascial necrosis with an obliterative endarteritis leading to further tissue necrosis with or without the involvement of underlying muscle [3, 8]. This condition can affect all age groups (with a mean age of 50) and has a male predominance [9, 10]. The mortality rate from this infection ranges from 0 to 67% [2]. The outcome is usually fatal if there is no early recognition or extensive surgical debridement upon initial diagnosis. Here, we report a case of extensive perineum necrotizing fasciitis that spread to near the anus. NPWT therapy was applied after STSGs to help heal the wound without faecal diversion.

When the FG spreads to near the anus, faecal diversion has been considered for several patients [1, 3] because the wound is vulnerable to contamination from excrement, which increases the wound extent and results in graft loss [6]. Second, the perineum is very mobile, and the shear stress to a skin graft prohibits healing and engraftment [11]. Furthermore, the irregular skin surface of the perineum prevents the vacuum leakproofing of negative pressure dressings, which is used to ensure that STSGs will exert even pressure across the wound; this leads to challenges during clinical treatment [12]. Simultaneously, colostomy often increases the patient’s economic burden and is unpleasant for the patient due to the associated psychological stress; the protracted course of the disease also increases patient burden [13].

Evidence has shown that NPWT combined with STSG can help wound healing [5].The skin from the scalp is easy to access and can be used on different parts of the body. In addition, when the wound defect is extensive and infectious, the skin from the scalp can be applied several times with a high possibility of remaining alive after transplantation. Therefore, we chose the scalp as the donor site. Because the wound had spread to the anus, a preoperative enema was used, an intra-operative iodoform gauze was used to fill the anus, the wound and the anus were covered by NPWT, and the post-operative non-residue diet was managed; together, these measures reduced the risk of graft loss caused by excrement contamination, improved STSGs survival, and avoided repeated surgical debridement and faecal diversion.

The mechanisms by which NPWT dressings facilitate various wound healing are clear. In this case, traditional comprehensive therapy was used to complete the wound bed preparation; then, we used NPWT only after STSGs. Infected wounds may require more frequent dressing changes; therefore, for cost reasons, the patient desired conventional treatment, which resulted in a longer hospitalization time. Although NPWT dressings and devices are more expensive than other wound-care products, a cost-effectiveness analysis showed lower treatment expenses. In a randomized controlled trial, Vuerstaek et al. demonstrated significantly shorter wound-preparation time and faster complete healing compared with the control group. The authors emphasized that the higher treatment costs of the control group were created by higher personnel costs and the longer hospitalization time due to slower healing [14]. However, we did not study enough cases to determine whether the preoperative non-usage of NPWT increased the hospitalization expenses.

At discharge, the patient was satisfied with the outcome. NPWT combined with STSGs to help heal the wound without faecal diversion or other complications was successful. We provided a method for surgeons to treat rare cases such as this, but further research is necessary to observe the efficacy of this method in a long-term follow-up study.

## Conclusions

This case showed that NPWT combined with STSGs to cover the whole wound and the anus without faecal diversion is safe and efficacious for healing extensive perineum necrotizing fasciitis that has spread to near the anus. However, the hospitalization time of the patient was relatively long because of the non-use of NPWT before STSGs. Not enough cases were available to compare the preoperative non-use of NPWT and determine whether the hospitalization expenses were higher. Meanwhile, more case series and randomized controlled trials are needed to better evaluate the treatment of this condition.

## Abbreviations

FG: Fournier gangrene
NPWT: Negative pressure wound therapy
STSG: Split thickness skin graft
